# The Interplay between Dengue Virus and the Human Innate Immune System: A Game of Hide and Seek

**DOI:** 10.3390/vaccines7040145

**Published:** 2019-10-10

**Authors:** Nicolas Tremblay, Wesley Freppel, Aïssatou Aïcha Sow, Laurent Chatel-Chaix

**Affiliations:** Institut national de la recherche scientifique, Centre Armand-Frappier Santé Biotechnologie, Laval, QC H7V 1B4, Canada; Nicolas.Tremblay@iaf.inrs.ca (N.T.); Wesley.Freppel@iaf.inrs.ca (W.F.); Aicha.Sow@iaf.inrs.ca (A.A.S.)

**Keywords:** dengue virus, innate immunity, viral evasion, interferon, MAVS, RIG-I, mitochondria

## Abstract

With 40% of the world population at risk, infections with dengue virus (DENV) constitute a serious threat to public health. While there is no antiviral therapy available against this potentially lethal disease, the efficacy of the only approved vaccine is not optimal and its safety has been recently questioned. In order to develop better vaccines based on attenuated and/or chimeric viruses, one must consider how the human immune system is engaged during DENV infection. The activation of the innate immunity through the detection of viruses by cellular sensors is the first line of defence against those pathogens. This triggers a cascade of events which establishes an antiviral state at the cell level and leads to a global immunological response. However, DENV has evolved to interfere with the innate immune signalling at multiple levels, hence dampening antiviral responses and favouring viral replication and dissemination. This review elaborates on the interplay between DENV and the innate immune system. A special focus is given on the viral countermeasure mechanisms reported over the last decade which should be taken into consideration during vaccine development.

## 1. Introduction

### 1.1. Disease Burden

Dengue is a neglected tropical disease caused by the dengue virus (DENV) that is transmitted to humans by *Aedes aegypti* or *Aedes albopictus* mosquitoes. In the recent years, it has become a public health concern, given that its global incidence has dramatically increased over the last 20 years and that no effective antiviral therapies are currently available. A dengue vaccine developed by Sanofi Pasteur, Dengvaxia, has been recently approved in over 20 countries around the globe. However, while the efficacy of this vaccine is not optimal for all DENV serotypes, its safety has been seriously questioned, especially for seronegative individuals. Epidemiological models estimate that about 390 million out of the 3.9 billion people living in endemic or epidemic areas contract the disease each year, making it the most prevalent arbovirus infection [1,2]. About 60 million infected individuals per year will develop symptomatic dengue fever, resulting in 10 to 20 thousand fatalities and 1.14 million Disability-Adjusted Life Years (DALY) [3]. This increase in the disease burden is not evenly distributed across the globe, with Latin America and the Caribbean ending up since 1990 with an increase in dengue-related DALY of about 250% or 4-fold above the global average and with Southeast Asia accounting for 596,000 DALY. This might be due to the simultaneous occurrence of multiple factors in these low-to-middle income emerging countries which affect the complex balance of DENV transmission dynamics, leading to a rapid endemic–epidemic cycle. For instance, this includes an optimal climate for *Aedes* life cycle, a rapid increase in population density in urban centres, and the concomitant circulation of all DENV serotypes (1–4) in one given restricted geographic area [4,5]. While there is an unmet medical need regarding strategies against dengue, the incidence of this disease is expected to geographically expand in the future considering that the arthropod vector is colonizing northern European and American territories with temperate climates.

### 1.2. Clinical Manifestation

DENV infection is characterized by a mixed clinical presentation that ranges from an asymptomatic disease to a mild febrile prodrome all the way to a severe hemorrhagic fever and shock syndrome. All infected individuals, asymptomatic or not, can transmit DENV to *Aedes* mosquitoes during a blood meal, making it difficult to precisely estimate the actual size of the reservoir at any given time. DENV infection severity is classified according to the World Health Organization (WHO) 1997 and 2009 guidelines [6]. Dengue “without warning signs” (or dengue fever) regroups at-risk individuals with fever and at least two of the following signs and symptoms: nausea/vomiting, rash, headaches, eye pain, muscle aches, joint pain, leukopenia, or positivity at the tourniquet test. Dengue “with warning signs of severe infection” (or dengue hemorrhagic fever) includes in addition to the previous signs and symptoms abdominal pain, persistent vomiting, ascites, pleural effusion, mucosal bleeding, lethargy or restlessness, hepatomegaly, and an increase in hematocrit paired with rapid decrease in platelet count. Lastly, severe dengue (or dengue shock) occurs when the infection leads to severe plasma leakage, massive bleeding, and multiple organ failures. As of now, the therapeutic arsenal against DENV infection is fairly limited and only consists of supportive care and intravenous fluid therapy.

From a host–pathogen interaction point of view, it is interesting to note that, in the majority of DENV-infected individuals, viremia is controlled by the innate and adaptive immune systems within three to seven days, independently of the clinical manifestation [7]. However, in some cases, the infection is not properly handled and symptoms aggravate. A higher peak in viral titres seems to be predictive of disease severity [8,9]. While the pathophysiology behind severe dengue is not fully understood, it is accepted that dengue hemorrhagic fever and severe dengue result from a cytokine-mediated pathology (cytokine storm) that occurs because of the unbalanced production of various soluble and short-lived immune factors, such as TNF-α, VEGF-A, IL-6, IL-8, IL-10, CCL2, and CXCL10, that are produced by immune cells [10].

### 1.3. Dengue Virus Life Cycle

DENV is an enveloped, positive-stranded RNA virus and belongs to the *Flavivirus* genus within the Flaviviridae family. Flaviviruses also include genetically related and/or medically relevant arthropod-borne pathogens such as yellow fever virus (YFV), Zika virus (ZIKV), West Nile virus (WNV), and Japanese encephalitis virus (JEV). The enveloped virion is constituted of prM/M (precursor membrane/membrane) and E (envelope) structural proteins at the surface while C nucleocapsid protein surrounds the 11 kilobase-long non-segmented viral RNA genome (vRNA). Following virus entry into the target cell, vRNA is translated into one polyprotein, which is subsequently processed by host and viral proteases to generate the structural proteins C, prM, and E and the seven nonstructural (NS) proteins NS1, NS2A, NS2B, NS3, NS4A, NS4B, and NS5. NS proteins are responsible for vRNA replication that occurs exclusively in the cytoplasm of infected cells. This involves the specific enzymatic activity of several NS proteins. The NS5 RNA-dependent RNA polymerase is responsible for vRNA synthesis and possesses a methyltransferase activity which is absolutely required for vRNA 5′ capping and 2′-*O*-methylation. The processing of the DENV polyprotein involves the serine protease activity of the NS2B/NS3 complex (or NS2B3). NS3 is also essential for vRNA synthesis and capping through its helicase, NTPase, and triphosphatase activities. Assembled virions bud into the endoplasmic reticulum (ER) and are taken in charge by the cellular secretion machinery. In the Golgi apparatus, they undergo a final round of furin-mediated maturation and acquire infectivity before their release via exocytosis [11,12,13]. In order to create a cytoplasmic environment that can sustain an optimal life cycle, DENV, much like other flaviviruses, induces within the infected cell the biogenesis of specific membranous replication organelles or replication factories (RF) with unique morphologies [14,15]. These RFs are formed by altering the curvature and composition of cellular endomembranes. They have been characterized as ER-derived ultrastructures that include (1) vesicle packets (VP) which result from membrane invaginations and contain the vRNA replication components; (2) convoluted membranes (CM) which are enriched in NS3, NS4A, and NS4B and of which the exact role(s) remain poorly defined; and (3) virus bags which contain immature virions organized in regular arrays [16,17,18]. In addition, DENV also manipulates the architecture of other organelles (such as mitochondria or peroxisomes) to modulate specific cellular functions, including innate immunity, in favour of replication (see below). While DENV is genetically simple with only 10 viral protein expressed, its life cycle relies on highly coordinated machineries that offer various points of intervention and research interests.

Following the entry of the virus inside the cell, it is rapidly sensed as a foreign “intruder” by the cell surveillance machinery, which triggers innate immunity. Strikingly, in response to these defence mechanisms, DENV has evolved to efficiently hide and mask its foreign molecular signatures, hence, avoiding the establishment of an antiviral environment that is detrimental for replication. In addition to these “cloak-and-dagger” operations, many viral proteins are able to directly hit the host antiviral machinery to shutdown innate immune signalling. Thus, antiviral immunity and DENV life cycle are dynamically interconnected events which balance each other; on one hand, the virus is trying to hijack the functions of the cellular machinery to its own benefit, while, on the other hand, the host factors are trying to control the viral infection while minimizing damages to cell homeostasis.

In this review, we elaborate on the interplay between DENV and innate immunity. More precisely, it brings into focus how DENV is detected inside an infected cell as well as how it can hide to avoid detection. A better understanding of how DENV circumvents and/or attenuates innate immunity can provide critical information for rational vaccine development.

## 2. Recognition of DENV by Pathogen Recognition Receptors

**Figure 1:**
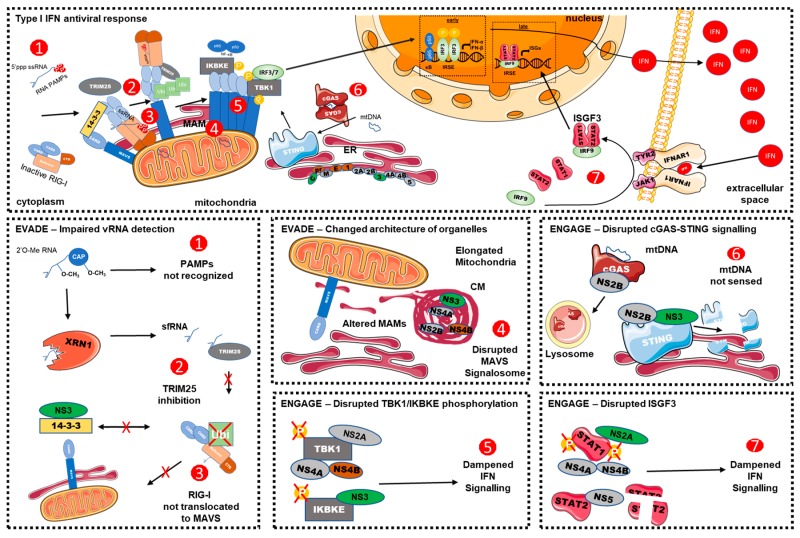
The sensing of Dengue virus by the innate immune responses and multiple evasion strategies to hinder the production of type I interferons (IFN).The upper panel shows the sensing of viral infection by RIG-I and cGAS. DENV-derived PAMPs are recognized by various pathogen recognition receptors like RIG-I. Upon recognition of pathogenic RNA, RIG-I is translocated to the mitochondria-associated MAVS adaptor protein with the help of the 14-3-3 and TRIM25 proteins. This induces the formation of MAVS aggregates that serve as an immune signalosome to activate through phosphorylation transcription factors IRF3 and NF-κB. In turn, these transcription factors translocate to the nucleus and elicit the transactivation of type I IFN. Once produced, type I IFN is secreted into the extracellular space and activates, in paracrine and autocrine fashions, the JAK-STAT pathway, leading to the amplification of the antiviral response via the activation of STAT1, STAT2, and IRF9, a transcription factor complex known as ISGF3. Infection also leads to mitochondrial stress and the release of mtDNA into the cytosol. mtDNA is recognized by cGAS, which activates STING, leading to the activation of IRF3/NF-κB and IFN production. DENV has developed various countermeasures to evade and/or engage the RLR and cGAS/STING pathways and to hinder innate immune signalling. These evasion mechanisms include (**1**) posttranscriptional modification of vRNA; (**2**) inhibition of 14-3-3ε or TRIM25 to hinder RIG-I activation; (**3**) MAVS aggregation; (**4**) alterations in endomembrane architecture to disrupt the MAVS signalosome; (**5**) the inhibition of TBK1 and IKBKE kinase activation, preventing the activation of transcription factors like IRF3; (**6**) the disruption of cGAS/STING signalling; and (**7**) the dampening of ISGF3 complex activation that is required for late innate antiviral responses.

Innate antiviral immunity is the first line of defence against viral pathogens and participates in the establishment of the adaptive immune response. It engages complex networks of proteins that are able to detect, control, and defeat viral threats. Virus detection is done by specialized proteins called Pattern Recognition Receptors (PRR), which can recognize various virus-specific conserved molecular signatures known as Pathogen-Associated Molecular Patterns (PAMP). This detection signal is transduced by adaptor proteins to ultimately activate various transcription factors (e.g., Interferon Regulatory Factors (IRF) 3 and 7) that drive the production of antiviral proteins including type I and type III interferons (IFN) (Figure 1, upper panel) [19,20,21,22]. Once secreted, these cytokines activate the Janus Kinase-Signal Transducer and Activator of Transcription (JAK-STAT) pathway, leading to the production of many Interferon-Stimulated Genes (ISG), which contribute to sustained paracrine and autocrine antiviral responses. Briefly, following the binding of type I IFN to the Interferon-α/β Receptors (IFNAR) 1/2, signalling through the JAK-STAT pathway results in the amplification of the antiviral response via the activation of STAT1, STAT2, and IRF9, a transcription factor complex known as IFN-stimulated gene factor 3 (ISGF3) [23,24]. All in all, innate antiviral immunity is a crucial cellular system that provides a quick reaction force against a given pathogen infection and can eventually lead to the establishment of a more focused yet costly effector response [25,26].

### 2.1. DENV vRNA-Recognition

The RIG-I-Like Receptor (RLR) pathway has been classically studied for its role in the detection of viral ribonucleic acids and involves Retinoic Acid-Inducible gene I (RIG-I or DDX58), Melanoma Differentiation-Associated protein 5 (MDA5), and Laboratory of Genetics and Physiology 2 (LGP2/DHX58) as cytosolic sensors of an infection. They are ubiquitously expressed and are able to sense specific viral RNA moieties such as short uncapped 5′ triphosphorylated single-stranded (ss) RNA, short double-stranded (ds) RNA, U/A-rich 3′ regions of viral RNA, or long dsRNA [27]. Upon binding to vRNA, RIG-I and MDA5 are activated and translocated to the surface of the mitochondria where they interact with an adaptor protein, Mitochondrial Antiviral-Signalling Protein (MAVS), via their respective caspase activation and recruitment domains (CARD) (Figure 1, upper panel). This triggers MAVS oligomerization and the formation of prion-like structures required to transduce the signal to downstream effector proteins. This signalling platform, often called MAVS signalosome is believed to bring together to the mitochondrial surface both upstream PRRs and downstream regulatory subunits (TRAF2, TRAF5, TRAF6, and NEMO) and protein kinases (TBK1 and IKBKE, also known as IKKε). Once activated, this signalosome orchestrates the phosphorylation of the transcription factors IRF3 and NF-κB, resulting in their nuclear translocation and the induction of type 1 IFN [28,29,30,31,32,33,34]. Remarkably, the activity of the MAVS regulome is tightly regulated by the morphodynamics of mitochondria as well as their contacts with the ER (see below) [35,36,37,38]. To sum up, mitochondria outer membrane is the scaffold of RLR signalling and, thus, plays an essential role in the initiation, the propagation, and the amplification of innate immunity against RNA viruses.

To date, there are several lines of evidence suggesting that that DENV is preferentially and directly sensed by the RLR pathway. An early study using RIG-I dominant-negative mutants showed that RIG-I is required to trigger IRF-3-dependent antiviral immunity against DENV in A549 lung cancer cells [39]. This observation was then recapitulated in various cell types (Huh7 hepatocarcinoma cells, endothelial cells, and primary monocytes) and experimental settings [17,40,41,42]. Combining affinity purification and next-generation sequencing, Chazal and colleagues recently showed that RIG-I is able to recognize 5′-ppp uncapped DENV vRNA, suggesting that detection of DENV nucleic acids occurs before the 5′-end maturation by capping is completed and that vRNA is afterwards hidden from PRR [43]. In stark contrast, the putative role of MDA5 in the detection of DENV nucleic acids remains unclear. Several groups have shown that silencing or interfering with MDA5 results in increased viral susceptibility and an altered innate immune response, but no clear mechanism of direct vRNA sensing has emerged despite this body of evidence. This might be due to the fact that all of these studies addressed the role of MDA5 in the early detection of vRNA. Strikingly, in the case of WNV, RIG-I and MDA5 have nonredundant roles at various stages of infection *in vitro* and *in vivo* [44]. Indeed, it was shown that MDA5 signalling to rely on the presence of WNV RNA PAMPs processed by cellular factors to become immunostimulatory but not on virion RNA that is initially uncoated upon viral entry. Overall, these studies support the view that RIG-I is a major player of the early detection of DENV RNA and of the initiation of the immune response. These further highlight that additional work is required to define the role of MDA5 during DENV infection.

Toll-like receptors (TLR) constitute another important family of PRR. They are located at the plasma membrane or within late endosomes and can sense a variety of PAMPs such as bacterial lipopolysaccharide, dsDNA, ssRNA, and dsRNA [45,46]. For DENV infection, TLR3 can recognize vRNA and its stimulation or overexpression dampens DENV replication *in cellulo* [47,48]. Both TLR and RLR pathways converge to MAVS and stimulate IRF3, NF-κB, and type I IFN production. While it remains unknown whether DENV stimulates TLR3 in vivo, a study in rhesus macaques showed that the combined agonist-mediated stimulation of both TLR3 and TLR7/8 following DENV infection resulted in a decrease in viral replication and enhanced pro-inflammatory responses [49]. This highlights the antiviral potential of the TLR3 pathway against DENV *in vivo*.

### 2.2. The DNA Sensing Pathway and DENV Infection

Converging to the same downstream effector molecules as the RLR pathway, that is, the TBK1/IRF3/IFN-β axis, the foreign DNA-sensing pathway relies on various intracellular DNA sensors, such as cyclic GMP-AMP Synthase (cGAS), which detect dsDNA danger signals from self or pathogen [50]. Upon binding, cGAS produces the second messenger cyclic GMP-AMP (cGAMP), which in turn activates Stimulator of Interferon Genes (STING). The engagement of the STING/cGAS pathway leads to the stimulation of TBK1 and ultimately to the production of type I IFN. Since its discovery, this pathway has been at the centre of many research interests in the fields of cancer, autoimmunity, inflammation, senescence, cell death, and viral infection, among others. Interestingly, while sensing DNA, the cGAS/STING pathway has been reported to be essential for the optimal innate immune response against RNA viruses [51,52,53]. Not surprisingly, several viruses such as influenza virus, hepatitis C virus, and coronavirus [52,54,55,56] have developed targeted evasion mechanisms to interfere with this pathway. The involvement of cGAS and STING in the innate immune response against DENV has become evident over the last few years, with several reports showing that DENV actually modulates this pathway (see below) [54,57,58,59]. While it remains unclear how a DNA-binding protein, like cGAS, can detect viral RNA, it was recently suggested that the sensing of DENV infection rather occurs indirectly. Indeed, virus-induced mitochondrial stress leads to the leakage of mitochondrial DNA (mtDNA) into the cytosol. It is subsequently sensed by cGAS, which in turn activates the type I IFN response [60]. More recently, it was shown that following inflammasome activation, secreted Interleukin (IL)-1β can induce mtDNA release, leading to the stimulation of the cGAS pathway and IFN production [61]. Considering that DENV infection triggers the activation of the inflammasome in human platelets and macrophages [62,63], it is plausible that this indirectly contributes to the mtDNA-dependent induction of early innate immunity.

Lastly, the emerging concept of a cross talk between the RIG-I/MAVS and cGAS/STING pathways is surely interesting to understand DENV biology. Indeed, there is an accumulating literature that shows that the RLR pathway is able to potentiate the cGAS/STING pathway and vice versa during RNA virus infection [64]. This seems to occur through the physical connection between RIG, MAVS, and STING during viral infection [51,65,66], the coactivation of both pathways [52,53,67], or a transcriptional feedback loops [66,68,69]. For flaviviral infections, a study has shown that STING potentiates the RLR signalling via the assembly of a RIG-I/MAVS/STING complex following JEV nucleic acid recognition [66]. In addition, another study in mice has shown that cGAS is required for the optimal RLR-dependent IFN response against WNV. Indeed, cGAS mice had a higher viral load and lower ISG expression, resulting in a higher mortality when compared to wild-type controls [53]. Future studies will be required to assess the role of the cross talk between the RIG-I/MAVS and cGAS/STING during DENV infection.

More generally, it will be required to investigate the exact nature, role, and distribution of the immunomodulatory molecules and signalling adaptors that are produced and expressed as a result of the pathogenesis of DENV infection. This will ultimately provide mechanistic insights on the exact contribution of the cGAS/STING pathway during DENV infection.

## 3. Viral Countermeasures

### 3.1. Interference with RLR-Dependent Signalling

DENV RNA synthesis and capping are believed to occur within the VPs since the viral replication dsRNA intermediate localizes within this ER-derived ultrastructure as shown by imaging studies using immunogold labelling and electron microscopy [16]. This confined environment is most probably important not only to concentrate the metabolites and proteins required for replication but also to exclude potentially inhibitory host factors. Considering this, it is tempting to speculate that neo-synthesized uncapped DENV RNAs “hide” from RIG-I in VPs although this model remains to be experimentally validated.

The replicated DENV RNA molecule possesses several intrinsic features that allow its evasion from detection by the innate immune system. These sensing interference strategies rely on two specific modifications of vRNA: its 2′-*O*-methylation and its partial degradation by host nucleases (Figure 1, bottom left panel). In addition to its RNA polymerase function, DENV NS5 has a methyltransferase (MTase) activity that generates the 5′ 7-methyl-guanosine cap and also methylates the 2′-*O*H position on the first nucleotide, forming a type 1 cap (m7GpppNm) structure [70,71]. The 2′-*O*-methylated vRNAs mimic cellular mRNAs, thus evading the host immune system [72,73,74,75,76] (Figure 1). Surprisingly, this mechanism is not necessarily strictly dependent on the capping of the vRNA since internal 2′-*O*-methylation can occur on vRNA lacking the 5′ cap structure during infections with DENV and other flavivirus such as WNV [72]. The 2′-*O*-methylation has been shown to be important to avoid coronavirus infection recognition by MDA5 in mouse and human cells. Indeed, a DENV2 mutant deficient in the 2′-*O* MTase activity (NS5 E217A mutation) leads to an attenuated viral spread in IFN-competent cells [73,74]. Consistent with this, it was later shown that the replication of the 2′-*O*-MTase mutant is attenuated at the very early stages of the life cycle, i.e. during the first 16 h of infection. As shown by a transcriptome kinetic analysis, this resulted in an increase in ISG transcription at the early time points postinfection with an enrichment in PRR genes (RIG-I, MDA5, and IFIT1). Altogether, these studies strongly support that DENV 2′-*O*-methylation aims to hide viral nucleic acids from early recognition by RLRs [76]. Importantly, a 2′-*O*-MTase mutant DENV2 strain was identified as a vaccine candidate since immunization with it conferred protection against subsequent DENV infection in mice and rhesus macaques [75]. However, it was proposed that exacerbated immune response might be due rather to a 2′-*O*-methylation-mediated evasion of the antiviral action of specific ISGs that enact downstream RLR and IFN signalling (see below).

Another vRNA-related evasion mechanism relies on the partial degradation of vRNA by host factors, resulting in the generation of the subgenomic flavivirus RNA (sfRNA), a small noncoding RNA (Figure 1). sfRNAs are produced as byproducts of an incomplete degradation of flaviviral RNA due to unique secondary structures in the 3′ UTR that causes XRN1, a 5′–3′ exoribonuclease, to stall and cut the vRNA prematurely [77,78]. Produced by all tested flaviviruses, the accumulation of biologically active sfRNA can contribute to virus-induced cytopathic effects [78,79]; to hijack mRNA processing machinery [80,81]; to promote optimal viral replication [82]; and most relevant here, to help the virus to circumvent antiviral signalling [83,84,85]. Indeed, in the case of DENV, one study showed that DENV sfRNAs are able to inhibit TRIM25 deubiquitylation by USP15 [86]. This prevents the K63 polyubiquitylation of RIG-I, its dimerization via its CARD domains; and its subsequent interaction with downstream adaptor protein MAVS, resulting in hindered interferon signalling [31,87,88]. On the whole, vRNA methylation and degradation into sfRNA consist of two evolutionarily conserved flaviviral strategies to counteract innate antiviral immunity.

In addition to interfering with RLR sensing of vRNA, DENV has developed other evasion strategies to inhibit the signalling cascade following RLR activation. Several DENV proteins modulate the pathway both upstream and downstream from MAVS. Notably, DENV NS3, through a conserved phosphomimetic RxEP motif, is able to prevent RIG-I translocation to the mitochondria by sequestering 14-3-3ε [89] (Figure 1). 14-3-3ε is required for efficient RIG-I/TRIM25 association and subsequent recruitment to mitochondria and interaction with MAVS [90]. Importantly, viruses expressing a mutated NS3 unable to associate with 14-3-3ε elicited a stronger immune response and replicated less efficiently in immune-competent cells [89]. Moreover, DENV NS4A is able to bind to MAVS CARD domains and to effectively prevent RIG-I/MAVS interaction through an unknown mechanism [91].

In order to sustain and maintain the immune synapse, MAVS needs to be physically accessible and at the proper position on the surface of the mitochondria; otherwise, antiviral signalling and response may not be optimal. Targeting this aspect of RLR-dependent innate immune signalling, DENV is also able to change the architecture of mitochondria in terms of morphodynamics and contacts with the endoplasmic reticulum (Figure 1). First, mitochondria show an elongated morphology in DENV-infected cells and make contacts with DENV convoluted membranes [17,92]. Interestingly, mitochondrial morphodynamics were reported to modulate antiviral signalling [35,36,37]. The morphology of mitochondria relies on an equilibrium between their fusion and fission regulated by dynamin-like GTPases. Briefly, these key players are mitofusins (MFN1 and MFN2) and Optic Atrophy 1 (OPA1), which mediate fusion (leading to elongation), and Dynamin-Related Protein 1 (DRP1), which mediates mitochondrial fission [93]. DENV-induced mitochondria elongation was attributed to NS4B since its overexpression alone recapitulated the phenotype. Furthermore, an inhibition of the phosphorylation-dependent activation of DRP1 was observed in both infected and NS4B-expressing cells, thus favouring mitochondrial elongation over fission [17].

Second, DENV infection resulted in a drastic disruption of the contacts between mitochondria and the ER (ERMC) [17]. Interestingly, the residual Mitochondria-Associated Membranes (MAM) appeared to be connected to CMs, suggesting that the biogenesis of this DENV RF substructure was responsible for ERMC disruption. Notably, MAMs are important for normal RLR signalling since they favour the tethering of MAVS required for optimal antiviral signalling [35,36,37,38]. This suggests that the alteration of the reticulo-mitochondrial interface by DENV would contribute to the dampening of RLR signalling. In strong support to this, upon enforced mitochondria elongation via DRP1 expression knockdown in DENV-infected Huh7 cells, RIG-I was barely recruited to the MAMs, correlating with a dampened type I and III IFN mRNA transcription and an increased viral replication. This highlights the functional interplay between mitochondria morphodynamics and contact with ER. In another study, Yu and colleagues showed that DENV NS2B3 protease cleaves MFN1 and MFN2 [94]. Interestingly, MFN1 overexpression-mediated hyperfusion of mitochondria in the perinuclear region resulted in a decreased viral replication. Moreover, independent expression knockdown of MFN1 or MFN2 led to different phenotypes in viral replication, IFN-β induction, and cell death, illustrating the divergent roles of mitofusins despite their similarity. This is consistent with the different assumed functions of MFN1 in docking and fusion of the mitochondria and of MFN2 in the stabilization of the interactions between mitochondria [95,96]. Furthermore, MFN2 localizes to MAMs and as an ER-resident protein tethers mitochondria to ER through homodimerization and heterotypic interactions with MFN1 [97]. This suggests that DENV might alter ERMCs by directly targeting MFN2 through NS2B3 protease activity without necessarily impacting on mitochondria fusion and elongation. Overall, these studies support the model that antiviral signalling is dependent on various spatiotemporal events and that alterations of the mitochondrial morphology and cytosolic interface regulate MAVS signalosome function. This concept of viral subversion of mitochondrial morphodynamics is not unique to DENV since related strategies have been observed for other viruses such as hepatitis B virus, hepatitis C virus, Severe acute respiratory syndrome-related coronavirus (SARS–CoV), and alphaherpesvirus [98,99,100,101].

In addition to ER and mitochondria, DENV impairs the morphology of another membranous organelle functionally linked to MAVS-related signalling. Indeed, DENV as well as WNV and ZIKV induce a loss of cellular peroxisomes [102,103], which contain MAVS at their surface and constitute a transduction platform for innate signalling [104,105,106]. Notably, the expression knockdown of the peroxin Pex19, which is essential for peroxisome biogenesis [107], led to a significant decrease in type III IFN gene transcription following the activation of RLR signalling with a synthetic dsRNA [102]. This highlights the importance of peroxisome integrity in innate immune signalling and the benefit for DENV of depleting them upon infection. Overall, these changes in cellular endomembrane morphology are believed to create a cytoplasmic microenvironment in which viral proteins are able to engage cellular host factors that are crucial to mount an adequate antiviral response.

Finally, downstream of MAVS, DENV is also able to evade RLR signalling by interfering with the activity of effector proteins (Figure 1). Indeed, NS2A, NS2B3, and NS4B are able to block IRF3 phosphorylation by interfering with serine kinase activity of TBK1 and IKBKE, which leads to a suboptimal type I IFN response [108,109].

All these evasion strategies are consistent with the idea that DENV physically targets the MAVS signalosome in its entirety when it brings together upstream and downstream factors to the cytosolic side of the mitochondria. Interestingly, several DENV proteins involved in this co-opting, namely NS2B3, NS4A, and NS4B, all interact and partly localize within CMs [16,17,18,110,111,112,113]. Furthermore, CMs make with elongated mitochondria and there is strong evidence that they originate from MAMs, an important compartment for MAVS-dependent signalling [17]. Altogether, this supports a model in which CMs constitute a “hijacking” unit, which targets the whole MAVS signalosome at multiple levels. Rather than directly regulating vRNA replication, this viral substructure would instead “shutoff” potentially antiviral responses to infection, including early innate immunity, hence creating a cytoplasmic environment favourable to viral replication. The close spatial relationship between CMs and mitochondria would help to “trap” host targets inside CMs or to concentrate them near viral proteins in order to very efficiently downregulate the MAVS signalosome and maximize viral evasion. Nevertheless, this model requires experimental challenging.

### 3.2. Interference with the cGAS/STING Pathway

DENV also has the capacity to directly interfere with IFN induction that is triggered by the activated cGAS/STING pathway as a result of the release of mtDNA into the cytoplasm following DENV infection [114] (Figure 1). Indeed, DENV NS2B marks cGAS for lysosomal degradation and DENV NS2B-NS3 protease cleaves STING to suppress type I IFN induction [54,58,114,115]. Interestingly, DENV NS2B3 cannot process either mouse or nonhuman primate STING orthologs, suggesting that this pathogen has evolved towards an optimal pathogenicity in its natural hosts [54,59]. Very recently, it has been reported that DRP1-mediated mitochondrial fission results in mitochondrial stress and the release of mtDNA in the cytosol [116]. Considering this, it will be interesting to investigate in the future whether DENV-induced mitochondrial elongation through DRP1 inhibition actually dampens not only RIG-I signal but also the activation of the STING/cGAS pathway and/or its cross talk with RLR signalling. In the same line of ideas, STING is a resident protein of MAMs [117], and the alteration of these structures during DENV infection might contribute to targeting STING to NS2B3-rich CMs for degradation or spatiotemporal sequestration.

### 3.3. Interference with the IFN Signalling

In addition to the primary virus sensing pathway, DENV is also able to target several components of the downstream interferon-induced JAK-STAT signalling cascade that normally leads to the sustained expression of various ISGs following the secretion of IFN-α and -β (Figure 1). Early *in vitro* assays demonstrated that DENV NS4B is able to prevent STAT1 activation by a mechanism conserved in WNV and YFV that is yet to be elucidated [118,119]. However, it can be rationalized that STAT1 inhibition most likely occurs either through prevention of its activation or by its dephosphorylation or by degradation of activated STAT1. On the matter of another STAT protein, DENV NS5 is able to bind STAT2 and to promote its E3 ubiquitin ligase UBR4-dependent degradation. This results in a drastic decrease of STAT2 basal expression level and, hence, to a hindered JAK-STAT signalling [120,121,122]. Interestingly, this STAT2-related evasion strategy is also conserved in other flaviviruses, such as ZIKV. Indeed, ZIKV is able to bind to STAT2 and to promote its proteasomal degradation but in an UBR4-independent manner [123,124].

### 3.4. Interference with Other Mechanisms

In addition to its role in STAT2 degradation, DENV NS5 protein is also able to interfere with ISG expression. First, both DENV and ZIKV NS5 selectively inhibit ISG expression by binding to and antagonizing the transcription factor PAF1C [125]. Second, DENV NS5 hijacks core components of the spliceosomal machinery, namely CD2BP2 and DDX23, resulting in an alteration of isoform abundance and stoichiometry of many antiviral factors such as RIG-I, ISG15, or IL-8, hence contributing to an environment favourable to viral replication [126]. At the posttranscriptional level, sfRNA has been shown to interfere with the translation of ISGs. Indeed, DENV sfRNA associates with the host RNA-binding proteins G3BP1, G3BP2, and CAPRIN1, which, as a result, are unable to stimulate as efficiently the translation of many ISGs such as PKR and IFITM2 for instance [127]. Consistently, sfRNA binding to these host factors protects DENV replication from IFN-β treatment. Interestingly, the 2′-*O*-methylation mechanism described above as a way to downregulate PRR early IFN response is also able to interfere with the functions of specific ISGs. Indeed, the replication of the NS5 E217A 2′-*O*-MTase DENV2 mutant is significantly more sensitive to IFN-β treatment than wt virus [75], demonstrating that this modification may contribute to immunity evasion downstream of PRR signalling. More specifically for DENV but also for WNV, poxvirus, coronavirus, and JEV, the 2′-*O*-methylation of vRNA cap enables the viral nucleic acids to be marked as “self-RNA” and to evade from the antiviral function of IFN-Induced Protein with Tetratricopeptide Repeat (IFIT) proteins [128,129,130,131]. Notably, IFIT1 was shown to associate with non-methylated RNAs with a higher affinity than the other IFITs. It was proposed that translation initiation factor eIF4E and IFIT1 compete for cap binding [129]. Hence, IFIT1 senses virus infection through the binding to foreign RNAs lacking 2′-*O*-methyls and inhibits the translation of viral RNA via the displacement of eIF4E. Consistently, IFIT1 overexpression in HEK293-DC-SIGN cells significantly decreases DENV2 replication in contrast to other IFITs [75]. In the case of WNV, the pathogenesis of the E218A MTase mutant is greatly attenuated in wt mice. Very interestingly, intracranial infection of *Ifit1* knockout C57BL/6 mice with the E218A MTase mutant WNV restored lethality, highlighting the critical role of this IFIT in the sensing of the “2′-*O*-methyl-free” vRNA *in vivo* [128]. Considering the genetic and biological proximity with WNV, it is tantalizing to speculate that DENV RNA is able to evade IFIT antiviral activity *in vivo* by mimicking cellular mRNAs through the addition of 2′-*O*-methyls, one of the most common posttranscriptional modifications of RNA allowing vRNA to hide in plain sight among host mRNAs.

As explained above, DENV sfRNA targets innate immune signalling in human cells at multiple levels (i.e. RIG-I activation and ISG expression). Of significant interest, several recent studies have highlighted the important role of sfRNA in the dissemination of DENV among infected insects [81,132,133,134]. In fact, the DENV and YFV subgenomic RNAs are able to interfere with the *Aedes* mosquito innate system as well by targeting both Toll receptor and RNA interference (RNAi) pathways to favour optimal replication. In the mosquitoes, viral double-stranded RNA is processed into small siRNAs by RNase III DICER and is processed into viral siRNAs (vsiRNAs) by AGO2, which are able to target viral RNA for silencing [135,136]. This shows that sfRNA plays two distinct roles, albeit using the same cellular factors, in the evasion of the innate immune response in mammalian hosts or arthropod vectors. This concept of different roles of host factors between the mammalian hosts or arthropod vectors will most likely be explored in depth in the future considering that a recent system biology study identified more than 45 shared pathway interactions between DENV–human and DENV–mosquito interactomes in the context of innate immune evasion and viral pathogenesis [125].

## 4. Importance for Vaccine Development

Multiple tetravalent vaccine candidates are being developed. These include live-attenuated vaccines, whole virus inactivated vaccines, protein-based vaccines, chimeric vaccines, and mRNA-based and synthetic virus-like particle vaccines [137,138,139,140,141,142]. Among these vaccines, Dengvaxia (also called CYD-TDV), developed by Sanofi Pasteur, is the only one licensed for use in about 20 dengue-endemic countries in Asia, Latin America, and Oceania as well as in Europe. CYD-TDV is a tetravalent dengue chimeric live-attenuated vaccine which is based on the YFV 17D strain as a backbone. This vaccine is typically indicated for individuals aged from 9 to 45 years living in an endemic country and, hence, is not accessible to the population that is the most at risk to develop severe dengue-related symptoms. Unfortunately, the vaccine confers less protection against serotypes 1 and 2 than with serotypes 3 and 4 [143,144]. Importantly, CYD-TDV efficacy was higher than pooled estimates in seropositive individuals but extremely low for seronegative children with risks of severe complications due to the vaccine. As a probable consequence of this, a global vaccination campaign in schools of the Philippines has resulted in the death of many children and the suspension of the program by the Department of Health in late 2017 [145]. As such, a pre-vaccination screening of previous exposure is now recommended prior to vaccination [146]. Lastly, a study shows that the CYD-TDV-elicited memory response decreases over time, as measured by low antibody titres in blood samples 5 years following an initial three-dose vaccine regimen. This suggests that a booster dose might be needed for sustained long-term immunological protection [147].

The development of new dengue vaccines which are safe and highly efficacious against all serotypes faces many challenges, including some involving the interplay between the virus and the immune system. Seminal studies by Albert Sabin, dating from the WWII era [148], have demonstrated that volunteers exposed to DENV showed long-lived homotypic immunity but short-lived cross-protection against viruses of a different serotype. However, the mechanisms behind these observations are still not fully understood and prospective studies at the population level show that these conclusions cannot be generalized to inform rational vaccine design [149]. While a majority of DENV infections go unnoticed, some infected individuals progress along the disease spectrum and develop dengue hemorrhagic fever or dengue shock. Although no consensus exists about the pathophysiology behind this progression, it is most likely due to host-specific factors related to the presence of preexisting immunity such as antibody-dependent enhancements (ADE) and immune-mediated cytokine storm related to the antigenic sin phenomenon that occurs even past the peak in viremia in symptomatic individuals [10,150]. Indeed, a second heterologous DENV infection is expected to elicit a strong memory recall of DENV-specific T and B cells that will produce an enhanced level of cytokines and neutralizing antibodies. However, this adaptive immune response might not lead to the control of viremia but rather quite the opposite, resulting in an antibody-dependent enhanced infection (virus opsonization and increased replication), mast cell activation (vascular permeability), and cellular cytotoxicity (cytokine storm) [151]. With this in mind, an effective antiviral therapy would need to be taken as a prophylactic treatment and would most likely be highly expensive given the number of individuals living in endemic areas. Thus, the only way to eradicate dengue worldwide would be to develop a safe, effective, and pan-serotypic vaccine that confers long-term sterilizing immunity.

In summary, while CYD-TDV constitutes a safe and efficacious tool in the therapeutic arsenal against DENV when properly administered, it is plagued by shortcomings and controversies and does not completely meet the need for an effective dengue vaccine, especially in seronegative individuals. Thus, many challenges remain to be addressed to develop the “holy grail” of DENV vaccines and a better understanding of host–pathogen interactions is warranted to set up new vaccinal strategies or relevant animal models. In the following section, we discuss three examples illustrating how innate immunity research can inform vaccine design.

### 4.1. DENVΔ30 Recombinant Dengue Virus

The DENVΔ30 is a live attenuated vaccine candidate that was engineered by deleting 30 nucleotides of the 3′UTR vRNA using reverse genetics and by subsequently creating modified dengue viruses for all four serotypes [152,153]. Proof-of-concept studies showed that, despite attenuation, the DENVΔ30 is highly immunogenic in both humans and rhesus monkeys for all tested serotypes and confers protection upon homologous rechallenge [153,154,155,156,157,158,159,160,161]. In addition, DENVΔ30 appears to be less virulent in mosquitoes, limiting the risk of transmission from a vaccinated human to the *Aedes* vector [162]. For long, the molecular mechanism conferring attenuation of this vaccine candidate was largely unknown. However, it was shown that DENV4Δ30-infected cells accumulated less sfRNA, resulting in an increased sensitivity to type I IFN [163]. Further studies will be required to assess if other evasion mechanisms are in play in DENVΔ30 attenuation to fully understand how this strain is able to create a balanced environment that is favourable to antigen presentation and cytokine production. However, given the reported roles of sfRNA in both IFN production and response (see above), it is likely that the attenuation of DENVΔ30 results from an increased innate immune response.

### 4.2. 2′-*O*-methyltransferase-Deficient Dengue Vaccine

As explained above, 2′-*O*-methylation is a modification that allows the vRNA to mimic cellular mRNAs and to evade the host innate immune system. Using reverse genetics, Züst and colleagues have generated DENV clones from serotypes 1 and 2 that harbour a single point mutation in the conserved KDKE motif of the NS5 methyltransferase catalytic site. These mutants (E216A for DENV1 or E217A for DENV2) are impaired in 2′-*O*-methylation but are still able to perform the N7-methylation of the cap. Importantly, they are more susceptible to IFN treatment or human IFIT1 overexpression than wild-type DENV, which is consistent with the idea that they are impaired in IFIT inhibition. Importantly, treatment of mice competent for T-cell responses with these viruses alone or in serotype combination conferred protection against a subsequent infection with a lethal mouse-adapted DENV strain. Remarkably, a single low dose of the DENV2 E217A mutant in monkeys was enough for a complete seroconversion and protection [75]. This approach was also applied to develop a JEV vaccine candidate. A E218A mutant JEV was attenuated and more sensitive to a type I IFN treatment as well [164]. A single dose of this live attenuated vaccine was sufficient to elicit a strong humoral response that conferred protection and survival following heterologous rechallenge in BALB/c mice. Ongoing studies are now trying to implement this vaccine rational design towards the development of a tetravalent, non-chimeric vaccine with therapeutic relevance.

### 4.3. Immunocompetent Mouse Model for Denv Vaccine research

Vaccine development studies usually rely on large animal models during nonclinical and preclinical studies [165]. The use of small animal models such as mice usually requires genetic modifications that dampen their immune system in order to increase viral permissiveness and, thus, do not reflect the reality of an infection in the natural host with an intact immune response. In most of the cases, *in vivo* DENV studies classically involve immunodeficient mouse strains such as AG129 which do not express IFN-α/β and IFN-γ receptors. These mice are highly susceptible to DENV infection and generally die within two weeks after injection [166,167,168]. Following advancement of the knowledge about host–pathogen interactions and the advent of novel technologies (e.g., by CRISPR/Cas9 gene editing), a paradigm shift can be anticipated. As an example of this, using a gene knock-in approach and a mouse-adapted virus strain, Gorman and colleagues recently developed an immunocompetent transgenic mouse model of ZIVK infection by replacing the mouse STAT2 by the human STAT2 [169]. This genetic engineering was conceptually made possible thanks to previous studies which showed that ZIKV induces the degradation of human STAT2 but not of the mouse ortholog [123,124,170]. While it will be interesting to see if the use of humanized immunocompetent mouse model can be expanded to DENV studies, it definitely opens up many future research opportunities.

## 5. Conclusions and Perspectives

DENV research will most likely remain a challenging and exciting field in times to come. In recent years, DENV infection has taken a front seat in the realm of global health concerns as it spreads outside the Western Pacific region and is now threatening more than 4 billion individuals. With no effective antiviral therapies or prophylactic vaccine, DENV is expected to cause disease and to inflict harm that amounts to more than 1.14 million DALY. As extensively discussed in this review, DENV is well equipped to evade the innate antiviral immune system through various antagonizing functions of viral NS proteins and RNA. From being able to hide in plain sight due to the 2′-*O*-methylation of the viral genome and to the disruption of MAVS signalosome activity and of the IFN signalling, it can be acknowledged that, despite a simple viral genome organization, DENV pathogenesis relies on a complex network of host–pathogen interactions. It is conceivable that better understanding this complexity will contribute to solving the puzzle of dengue control and/or eradication. Indeed, the interplay between DENV and the immune system can lead to rational vaccine design by providing attractive targets for antigen and adjuvant development and by filling the gaps in knowledge regarding how the innate immune system can be harnessed to support a long-lasting protective immune response.
